# Juvenile Hormone Analogues Reduce the Expression of a Fatty Acid-Binding Protein Involved in Lipid Accumulation in the Migratory Locust *Locusta migratoria*

**DOI:** 10.3390/insects17070664

**Published:** 2026-06-25

**Authors:** Tian Miao, Zige Wang, Min Peng, Jinchao Chen, Dengbo Li, Yuemin Ma

**Affiliations:** 1 College of Life Science, Shanxi University, Taiyuan 030006, China; miaotian@sxu.edu.cn (T.M.); wangzige1@sxu.edu.cn (Z.W.); pengmin@sxu.edu.cn (M.P.); chenjinchao@sxu.edu.cn (J.C.); lidengbo@sxu.edu.cn (D.L.); 2 School of Synthetic Biology, Shanxi University, Taiyuan 030006, China

**Keywords:** *Locusta migratoria*, *Mg-FABP*, lipid metabolism, fatty acid

## Abstract

Juvenile hormone (JH) analog insecticides are widely used in pest management because of their ability to disrupt insect growth and metamorphosis; however, the molecular mechanisms linking endocrine disruption to metabolic dysregulation remain incompletely understood. In addition to their established roles in diapause and developmental regulation, JH signaling pathways have also been implicated in carbohydrate and lipid metabolism. In the present study, we investigated the effects of two JH analogs, pyriproxyfen and hydroprene, on the migratory locust, *Locusta migratoria*, with particular emphasis on lipid metabolic regulation and the function of midgut-enriched fatty acid-binding protein gene (*Mg-FABP*). Bioassays were performed to evaluate insecticidal activity, and transcriptomic analyses were conducted to identify differentially expressed genes associated with endocrine signaling and lipid metabolism. Functional characterization of *Mg-FABP* was further performed using RNA interference (RNAi) and Oil Red O staining assays. In addition, the tertiary structure of LmMg-FABP was predicted using AlphaFold 3, and molecular docking analyses were carried out to investigate its interactions with fatty acid ligands. Both pyriproxyfen and hydroprene caused approximately 70% mortality in locust nymphs and induced significant transcriptional changes in pathways related to hormone signaling and lipid metabolism. Transcriptomic analysis revealed pronounced downregulation of *Mg-FABP* following JH analog exposure. RNAi-mediated silencing of *Mg-FABP* significantly reduced lipid droplet accumulation in the fat body, indicating that *Mg-FABP* plays an essential role in lipid transport and metabolic homeostasis in *L. migratoria*. Structural analyses further demonstrated that LmMg-FABP possesses a conserved tertiary structure highly similar to FABP homologs from other insect species. Molecular docking identified key amino acid residues involved in fatty acid binding and suggested that hydrophobic interactions are critical for ligand stabilization within the binding cavity. Collectively, our findings demonstrate that pyriproxyfen and hydroprene disrupt insect development not only through endocrine imbalance but also through perturbation of Mg-FABP-associated lipid metabolic pathways. This study provides new mechanistic insight into the coordinated interaction between hormonal signaling and lipid metabolism during JH analog exposure and identifies FABP-mediated lipid transport as a potential molecular target for the development of more selective insect growth regulators.

## Simple Summary

Juvenile hormone analogues (JHAs) are widely used insect growth regulators that interfere with insect development and metamorphosis. However, the molecular mechanisms underlying their toxicity remain incompletely understood. In this study, we investigated the effects of two JHAs, pyriproxyfen and hydroprene, on hormonal regulation and lipid metabolism in the migratory locust (*Locusta migratoria*). Transcriptomic analyses revealed that JHA exposure disrupted the balance between juvenile hormone and ecdysteroid signaling pathways and significantly altered the expression of genes involved in molting and development. In addition, lipid metabolism-related pathways were strongly affected, particularly the fatty acid-binding protein gene *Mg-FABP*, which showed marked downregulation after treatment. Structural and molecular docking analyses further demonstrated that LmMg-FABP possesses a conserved lipid-binding structure capable of interacting with multiple fatty acid ligands. These findings suggest that JHAs not only disrupt endocrine signaling but also impair lipid transport and metabolic homeostasis. This study provides new insight into the molecular mechanisms of JHA toxicity and identifies FABP-associated lipid metabolism as a potential target for the development of more selective insect growth regulators.

## 1. Introduction

Juvenile hormone (JH) is a sesquiterpenoid hormone that plays indispensable roles in regulating insect development, metamorphosis, reproduction, and behavior. Synthesized by the corpora allata, JH coordinates key life-history transitions by maintaining larval status, modulating molting processes, and regulating reproductive maturation [1,2]. At the molecular level, JH signaling is primarily mediated through its intracellular receptor Methoprene-tolerant (Met), which, upon ligand binding, forms a complex with Taiman (Tai) and activates downstream transcription factors such as Krüppel homolog 1 (Kr-h1), thereby orchestrating gene expression programs essential for development and reproduction [3,4]. Disruption of this finely regulated hormonal system can lead to severe developmental abnormalities, impaired reproduction, or lethality, underscoring the critical importance of JH homeostasis in insect physiology.

Exploiting this vulnerability, juvenile hormone analogues (JHAs) have been widely developed as insect growth regulators (IGRs) for pest management. Compounds such as pyriproxyfen and hydroprene mimic the biological activity of endogenous JH, thereby interfering with normal endocrine signaling and preventing successful metamorphosis or reproduction [5,6,7,8]. These compounds typically exhibit high selectivity toward immature stages and relatively low toxicity to vertebrates, making them attractive alternatives to conventional insecticides. For example, hydroprene has been extensively used in stored-product pest management, where it prolongs developmental duration, increases mortality, and inhibits adult emergence in several pest species [9]. Similarly, pyriproxyfen effectively suppresses metamorphosis and reproduction across a broad range of insect taxa by acting as a potent JH mimic. Even at relatively low concentrations, JHAs such as pyriproxyfen can disrupt larval development and inhibit adult emergence, particularly when used in combination with neurotoxic agents, supporting their utility in integrated pest management programs [10].

At the mechanistic level, JH analogues exert their effects primarily through activation of the JH signaling pathway. Pyriproxyfen has been shown to directly bind the Met receptor, mimicking endogenous JH and triggering downstream transcriptional cascades [3]. Consequently, exposure to pyriproxyfen often results in the upregulation of JH-responsive genes such as Kr-h1 and disruption of normal developmental timing [11]. Trace exposure to pyriproxyfen induces pronounced morphological damage in the posterior silk gland of *Bombyx mori* larvae, including shrinkage, vacuolization, and fragmentation, accompanied by activation of autophagy and apoptosis pathways. Transcriptomic and molecular analyses further indicate that pyriproxyfen disrupts endocrine signaling by activating JH-related genes while suppressing 20-hydroxyecdysone signaling, thereby promoting the expression of autophagy and apoptosis-related genes in the silk gland [12]. In parallel, JH analogues frequently antagonize ecdysteroid signaling, another key hormonal axis governing molting and metamorphosis. For instance, pyriproxyfen exposure suppresses ecdysone synthesis and signaling, leading to developmental arrest, abnormal metamorphosis, and reduced reproductive capacity in insects such as *Bombyx mori* [13,14]. Starvation suppresses juvenile hormone esterase (JHE) activity and *jhe* transcription, increases mortality, and reduces the molting rate from the third to fourth instar in *Plutella xylostella*, whereas treatment with 500 mg/L pyriproxyfen delays molting, completely inhibits pupation, and significantly elevates JHE activity at 48 h and *jhe* transcription at 24 and 48 h [15]. Pyriproxyfen also suppresses JH III titers in honey bees and disrupts normal pupation during larval metamorphosis, resulting in prolonged pupal duration. RT-qPCR analyses further show that pyriproxyfen enantiomers downregulate *AmCYP15A1* while upregulating *AmJHE*, *AmMet*, and *AmKr-h1* expression, indicating a JH-mimicking mode of action [16]. Similar endocrine-disrupting effects have also been reported for hydroprene, which prolongs larval development and interferes with normal metamorphic progression [5]. Collectively, these findings demonstrate that JH analogues disrupt insect development primarily through endocrine imbalance between JH and ecdysteroid signaling pathways.

Beyond classical endocrine pathways, accumulating evidence indicates that JH analogues can also perturb broader metabolic processes, particularly lipid metabolism. Lipids are essential components of insect physiology, serving as structural constituents of membranes, major energy reserves, and signaling molecules. Disruption of lipid homeostasis can therefore have profound consequences for growth, development, and reproduction. Recent metabolomic studies have revealed that exposure to pyriproxyfen and other IGRs significantly alters fatty acid composition and lipid metabolic pathways in insects [17]. In aquatic invertebrates such as *Daphnia magna*, pyriproxyfen induces tissue-specific lipidomic disturbances, including increased glycerolipid accumulation and altered glycerophospholipid composition [18]. These findings suggest that the biological effects of JH analogues extend beyond endocrine disruption to include metabolic dysregulation, which may contribute substantially to their overall toxicity.

The interplay between hormonal signaling and lipid metabolism is increasingly recognized as a key regulatory feature of insect physiology. Hormonal pathways such as ecdysone signaling coordinate nutrient allocation and metabolic remodeling during development. For example, the ecdysone-induced transcription factor *Eip75B* regulates fatty acid-binding protein (FABP) expression, forming a regulatory axis that integrates endocrine signals with lipid metabolism to maintain energy homeostasis [19]. Given that JH analogues can modulate both JH and ecdysteroid pathways, it is plausible that their effects on lipid metabolism are mediated, at least in part, through these interconnected hormonal networks.

Fatty acid-binding proteins (FABPs) are essential intracellular lipid chaperones that facilitate the transport, storage, and utilization of fatty acids and other hydrophobic ligands. These proteins are highly conserved across species and play fundamental roles in maintaining lipid homeostasis. In insects, FABPs have been implicated in diverse physiological processes, including development, reproduction, and energy metabolism. For instance, FABPs are required for efficient fatty acid transport and sustained flight in locusts, as their depletion severely impairs lipid utilization during prolonged flight [20,21]. In addition, FABPs contribute to lipid accumulation during diapause and regulate developmental processes in species such as *Drosophila melanogaster* and *Colaphellus bowringi* [22,23,24]. RNA interference (RNAi) analyses have demonstrated that FABPs are essential for lipid storage during early diapause, and suppression of these genes prevents female mosquitoes from accumulating sufficient lipid reserves for overwintering [22]. In *Bombyx mori*-derived BmN4 cells, dexamethasone treatment induces lipid accumulation, inhibits cell proliferation, and promotes cell aggregation, accompanied by upregulation of the fatty acid-binding protein gene *BmFABP1*, further supporting the role of *FABPs* in regulating lipid metabolism [25]. At the cellular level, FABPs also participate in intracellular lipid trafficking and cholesterol transport, highlighting their functional versatility [26].

Despite extensive studies on JH signaling and the widespread application of JH analogues such as pyriproxyfen and hydroprene, the potential involvement of *FABPs* in mediating the metabolic and toxicological effects of these compounds remains largely unexplored. In particular, the specific roles of *FABPs* in JH analogue-induced lipid metabolic disruption, as well as their integration within JH-regulated endocrine networks, are still poorly defined. Given the central importance of *FABPs* in intracellular lipid transport and homeostasis, elucidating their contribution to the action of JH analogues represents a critical gap in our understanding of insect physiology.

Therefore, this study aimed to investigate the functional roles of *Mg-FABP* in response to JH analogue exposure and to clarify their involvement in lipid metabolic regulation. To achieve this, we combined transcriptomic analysis, RNA interference, lipid staining, and molecular docking approaches to systematically characterize FABP-mediated lipid transport and its perturbation by JH analogues. In addition, structural prediction and ligand binding analyses were performed to further elucidate the interaction mechanisms between FABPs and fatty acids. Understanding how JH analogues influence lipid metabolism through FABP-associated pathways will not only provide new mechanistic insights into their modes of action but also advance our knowledge of the crosstalk between endocrine signaling and metabolic regulation in insects. Such findings are essential for improving the ecological risk assessment of insect growth regulators (IGRs) and may facilitate the development of more selective and environmentally sustainable pest management strategies.

## 2. Materials and Methods

### 2.1. Insect Rearing

Egg pods of *L. migratoria* were sourced from a commercial supplier (Locust Breeding Center, Cangzhou, China) through an online purchase. After hatching, locusts were maintained under controlled laboratory conditions with a 16:8 h light/dark cycle at 30 ± 1 °C and 50–60% relative humidity. Healthy and developmentally synchronized third-instar nymphs were selected for subsequent experiments.

### 2.2. Transcriptome Experimental Design, RNA Extraction, Library Construction, and Bioinformatic Analysis

#### 2.2.1. Insect Treatment and Sample Collection

Third-instar (1-day-old) *Locusta migratoria* nymphs were randomly assigned to three treatment groups: acetone (ACE, control; Sigma-Aldrich, St. Louis, MO, USA), pyriproxyfen (PPF; Supelco, Bellefonte, PA, USA), and hydroprene (HPN; Supelco, Bellefonte, PA, USA). Each insect received a 5 μL topical application on the abdominal tergite of an acetone-based solution, consisting of 10 μg/μL of the corresponding solution, whereas control insects were treated with 5 μL acetone. All treatments were performed under identical rearing conditions with three independent biological replicates per group. At 24 h post-treatment, midgut (MG) and fat body (FB) tissues were dissected from surviving insects, immediately frozen in liquid nitrogen, and stored at −80 °C until RNA extraction and transcriptome sequencing. At 5 days post-treatment, mortality was recorded in both the HPN- and PPF-treated groups to assess treatment effects.

#### 2.2.2. RNA Extraction and Quality Assessment

Total RNA was extracted using TRIzol reagent (Invitrogen, Carlsbad, CA, USA) according to the manufacturer’s instructions. RNA concentration and purity were determined using a NanoDrop 2000 spectrophotometer (Thermo Fisher Scientific, Waltham, MA, USA), while RNA integrity was evaluated using an Agilent 2100 Bioanalyzer (Agilent Technologies, Santa Clara, CA, USA). Only high-quality RNA samples were used for subsequent library construction and sequencing.

#### 2.2.3. cDNA Library Construction and RNA Sequencing

Poly(A)+ mRNA was enriched using oligo(dT) magnetic beads and subsequently fragmented under high-temperature conditions. The fragmented mRNA was used as a template for first-strand cDNA synthesis using reverse transcriptase, followed by second-strand cDNA synthesis. After end repair and A-tailing, sequencing adapters were ligated to the cDNA fragments. Target fragments were purified using Hieff NGS^®^ DNA (Yeasen Biotechnology, Shanghai, China) Selection Beads and amplified by PCR to generate the final sequencing libraries. Library quality and concentration were assessed prior to sequencing on the Illumina NovaSeq X Plus platform (Illumina, San Diego, CA, USA) to generate paired-end reads.

#### 2.2.4. Read Filtering and Genome Mapping

Raw sequencing reads were processed using fastp (v0.18.0) [27] to remove adapter sequences, reads containing more than 10% ambiguous nucleotides (N), and low-quality reads with more than 50% bases having Q-values ≤ 20. Ribosomal RNA (rRNA)-derived reads were identified and removed using Bowtie2 (v2.2.8) [28]. After quality control, high-quality clean reads were obtained and used for downstream analyses. All samples exhibited high sequencing quality, with clean read ratios ranging from 98.69% to 99.42% (Table S3). No biological replicates were excluded during the quality control process, and all samples were retained for subsequent analysis. The reference genome sequence and corresponding annotation files (GFF3, CDS, and protein sequences) were obtained from the InsectBase 2.0 database (http://v2.insect-genome.com/Organism/509, accessed on 12 June 2026), which is derived from the LocustBase dataset. The genome has a total size of 6476.7 Mb and contains 21,354 predicted protein-coding genes. The resulting high-quality clean reads were mapped to the reference genome using HISAT2 (v2.1.0) [29] with default parameters. All Supplementary Materials are provided as a single compressed archive accompanying the manuscript submission, including Supplementary Data S1–S3.

#### 2.2.5. Transcript Assembly and Quantification

Mapped reads were assembled using StringTie (v1.3.1) [30,31] in a reference-guided manner. Gene expression abundance was quantified as transcripts per million (TPM) using RSEM software (v1.2.19) [32].

#### 2.2.6. Sample Correlation and Principal Component Analysis

Correlation analysis among biological replicates was performed to evaluate the reproducibility and reliability of transcriptome data. Principal component analysis (PCA) was conducted using R packages (v1.20.0) to assess global transcriptional variation and sample clustering among different treatment groups.

#### 2.2.7. Differential Expression and Functional Enrichment Analysis

Differentially expressed genes (DEGs) were identified using DESeq2 [33]. Genes with |log2(fold change)| ≥ 1 and adjusted *p*-values < 0.05 following Benjamini–Hochberg correction were considered significantly differentially expressed. Gene Ontology (GO) [34] and Kyoto Encyclopedia of Genes and Genomes (KEGG) [35] enrichment analyses were performed using hypergeometric testing, and pathways or GO terms with false discovery rate (FDR) ≤ 0.05 were regarded as significantly enriched.

### 2.3. Phylogenetic Analysis of LmMg-FABP

Sequence similarity was analyzed using BLASTP (NCBI BLAST+ v2.17.0). The theoretical molecular weight (Mw) and isoelectric point (pI) were estimated using ProtParam (Expasy, https://web.expasy.org/protparam/, accessed on 12 June 2026). Multiple sequence alignment and phylogenetic analysis was carried out in MEGA 12 based on the neighbor-joining method with 1000 bootstrap replicates, using LmMg-FABP and homologous FABP amino acid sequences from other insect species. Conserved domains were identified using the NCBI Conserved Domain Database (CDD), and domain organization was visualized with DOG 1.0 [36].

### 2.4. RNA Interference and Bioassays

#### 2.4.1. dsRNA Synthesis and RNAi Procedure

Double-stranded RNA (dsRNA) was synthesized based on a previously reported method [37] using gene-specific primers containing T7 promoter sequences (Table S1). dsRNA targeting LmMg-FABP and GFP (control) was produced using the T7 RiboMAX™ Express RNAi System (Promega, Madison, WI, USA).

For gene silencing, 3 μL *dsLmMg-FABP* solution (6 μg dsRNA) was injected into the abdominal intersegmental membrane of one-day-old third-instar nymphs using a microinjector. Control groups were injected with an equal volume of *dsGFP*. Each treatment included three independent biological replicates, with eight individuals per replicate. At 48 h post-injection, fat body tissues were collected for RNA extraction and Quantitative real-time PCR (RT-qPCR) analysis to verify knockdown efficiency. RT-qPCR was conducted using a Bio-Rad real-time PCR system with SYBR Green Master Mix (TOYOBO, Tokyo, Japan), following previously described procedures with minor modifications [38]. EF1α was used as the reference gene, and relative transcript levels were calculated using the 2^−ΔΔCt^ method. Primer sequences are provided in Table S2.

#### 2.4.2. Functional Assays and Lipid Analysis

For lipid visualization, fat body tissues were embedded in OCT compound, sectioned at 10 μm thickness at −20 °C, and stained using a modified Oil Red O staining kit (Solarbio, Beijing, China) according to the manufacturer’s instructions. Images were captured using a Nikon E200 light microscope (Nikon, Tokyo, Japan). Triglyceride (TG) content was measured using the Applygen TG Assay Kit (Applygen, Beijing, China) according to the manufacturer’s instructions. Three biological replicates were performed.

### 2.5. Docking Simulation Analysis

The three-dimensional structure of LmMg-FABP was generated using AlphaFold 3 [39]. Model reliability was evaluated based on the predicted local distance difference test (pLDDT) and predicted template modeling (pTM) scores. A pTM score above 0.5 means the overall predicted fold for the complex might be similar to the true structure. The chemical structures of myristic acid (MYR) and palmitic acid (PLM) were obtained from the PubChem database. Structural quality of the predicted model was further validated using MolProbity [40] and Verify3D (https://www.doe-mbi.ucla.edu/verify3d/, accessed on 12 June 2026). Structural similarity searches were conducted with the DaliLite v.3 server [41] to identify homologous proteins. Protein–ligand complex models with MYR and PLM were generated using AlphaFold 3; the interface predicted template modeling (ipTM) measures the accuracy of the predicted relative positions of the subunits within the complex. ipTM values higher than 0.8 represent confident high-quality predictions. Binding interactions were analyzed and visualized using Discovery Studio 2021 (BIOVIA) and PyMOL (v4.6).

### 2.6. Statistical Analysis

All experiments were conducted with three independent biological replicates. Data are presented as mean ± SD. Data normality was assessed using the Shapiro–Wilk test, and homogeneity of variance was evaluated using an F test for two-group comparisons or the Brown–Forsythe test for multiple group comparisons. Statistical significance between two groups was assessed using an unpaired Student’s *t*-test (* *p* < 0.05, ** *p* < 0.01, ns, not significant), whereas multiple comparisons were performed using one-way ANOVA followed by Tukey’s multiple comparisons test. Different letters indicate statistically significant differences (*p* < 0.05), whereas identical letters indicate no significant difference (ns, *p* > 0.05). Analyses were performed using GraphPad Prism v8.0.2.

## 3. Results

### 3.1. Transcriptome Sequencing and Mapping Statistics

Following the observation of high mortality rates in locust nymphs after pyriproxyfen and hydroprene exposure (Supplementary Figure S1A,B), transcriptome sequencing analysis was carried out to further explore the associated molecular responses (Supplementary Figure S1C). RNA-seq generated high-quality transcriptomic data for all midgut (MG) and fat body (FB) samples under different treatments. After quality filtering, the number of clean reads per sample ranged from approximately 34.28 to 53.40 million. Genome alignment analysis showed that the overall mapping rates ranged from 80.80% to 88.58%, indicating high compatibility with the reference genome. Among the mapped reads, uniquely mapped reads accounted for 69.78–78.18% of total reads, whereas multiple mapped reads represented 9.53–12.67%. The proportion of unmapped reads ranged from 11.42% to 19.20% (Table 1). In addition, no obvious differences in mapping efficiency were observed among the acetone control (ACE), hydroprene treatment (HPN), and pyriproxyfen treatment (PPF) treatment groups, indicating good consistency among biological replicates. These results demonstrated that the sequencing datasets were of sufficient quality and reliability for subsequent differential expression and functional enrichment analyses.

### 3.2. Principal Component and Differential Expression Analyses Reveal Tissue Specific Transcriptomic Responses to JHA Treatments

**Figure 1:**
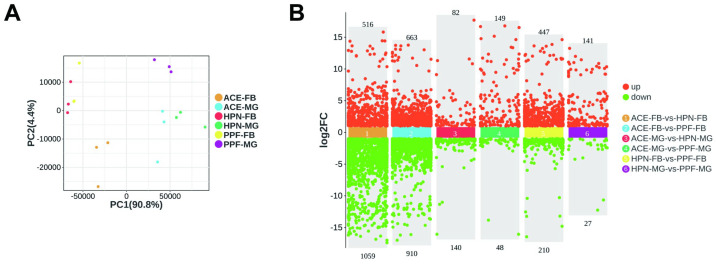
Transcriptomic variation and differential gene expression profiles in midgut (MG) and fat body (FB) tissues under juvenile hormone analogue treatments. (**A**) Principal component analysis (PCA) of transcriptomic datasets derived from MG and FB tissues under different treatments. PC1 and PC2 explained 90.8% and 4.4% of the total variance, respectively, showing clear separation among treatment groups. ACE, acetone control; HPN, hydroprene treatment; PPF, pyriproxyfen treatment; MG, midgut; FB, fat body. (**B**) Distribution of differentially expressed genes (DEGs) identified from pairwise comparisons. The x-axis represents the comparison groups, and the y-axis indicates log2 fold change (log2FC). Red dots indicate upregulated genes, and green dots indicate downregulated genes in each comparison. The bars above the x-axis represent upregulated genes and those below represent downregulated genes. The numbers displayed on the upper bars indicate the counts of upregulated genes, while those on the lower bars indicate the counts of downregulated genes in each comparison.

Principal component analysis (PCA) revealed clear differences among transcriptomic profiles derived from midgut (MG) and fat body (FB) tissues under ACE, HPN, and PPF treatments. PC1 and PC2 accounted for 90.8% and 4.4% of the total variance, respectively. Samples were clearly separated according to tissue type along PC1, indicating strong tissue-specific transcriptional characteristics (Figure 1A). Within each tissue, samples from different treatment groups formed distinct clusters, suggesting that both hydroprene (HPN) and pyriproxyfen (PPF) markedly affected gene expression patterns. Biological replicates clustered closely together in all groups, demonstrating high reproducibility and reliability of the RNA-seq data.

To further characterize treatment-induced transcriptional responses, differential expression analyses were conducted across pairwise comparisons between control and insecticide-treated groups in both tissues. In FB tissue, large numbers of differentially expressed genes (DEGs) were identified after both HPN and PPF treatments. Compared with the ACE control, 516 genes were upregulated and 1059 genes were downregulated in the ACE-FB vs. HPN-FB comparison, whereas 663 upregulated and 910 downregulated genes were identified in the ACE-FB vs. PPF-FB comparison (Figure 1B). In contrast, MG tissue exhibited relatively fewer transcriptional changes. The ACE-MG vs. HPN-MG comparison identified 82 upregulated and 140 downregulated DEGs, while the ACE-MG vs. PPF-MG comparison identified 149 upregulated and 48 downregulated DEGs (Figure 1B). Overall, substantially more DEGs were detected in FB than in MG under both JHA treatments, indicating that the fat body was more responsive to juvenile hormone analogue exposure.

### 3.3. GO Classification Analysis of Differentially Expressed Genes

**Figure 2:**
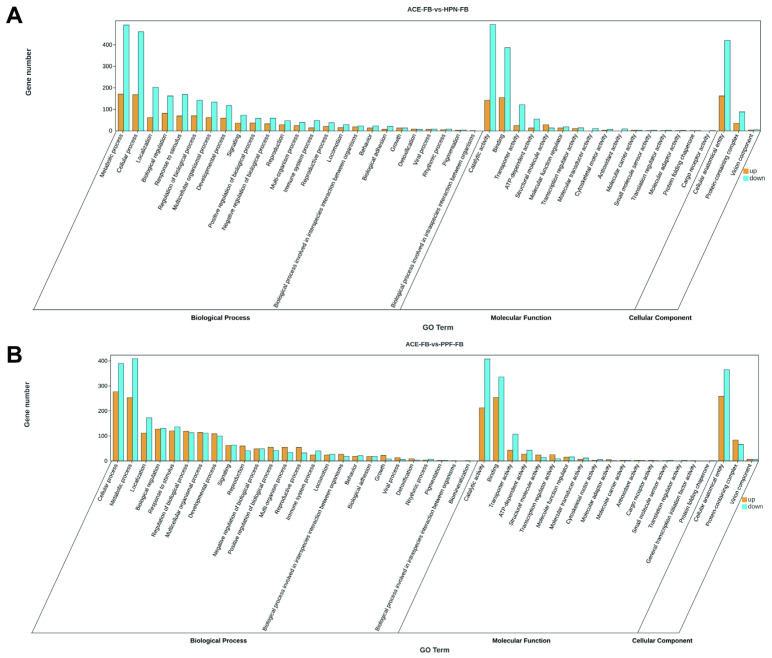
GO classification analysis of differentially expressed genes (DEGs) in fat body tissues following juvenile hormone analogue treatment. (**A**) GO classification of DEGs identified in the ACE-FB vs. HPN-FB comparison. (**B**) GO classification of DEGs identified in the ACE-FB vs. PPF-FB comparison. DEGs were categorized into three major Gene Ontology (GO) categories, including Biological Process (BP), Molecular Function (MF), and Cellular Component (CC). The x-axis represents the number of DEGs enriched in each GO term, while the y-axis indicates the corresponding GO functional categories. Orange bars represent upregulated genes, whereas blue bars represent downregulated genes.

GO classification analysis was conducted to characterize the functions of DEGs identified among different treatment groups (Figure 2A,B). The DEGs were assigned to three major GO categories, including biological process (BP), molecular function (MF), and cellular component (CC). In the BP category, most DEGs were enriched in metabolic process, cellular process, response to stimulus, and biological regulation. Development-related terms such as developmental process, reproductive process, and growth were also highly represented. Compared with hydroprene treatment, pyriproxyfen treatment resulted in a larger number of DEGs, particularly in the ACE-FB vs. PPF-FB comparison group. In the MF category, catalytic activity and binding were the most enriched terms, followed by transporter activity and ATP-dependent activity. These results suggest that juvenile hormone analogue exposure affected enzymatic activity, molecular binding, and transport-related functions. For the CC category, most DEGs were classified into cellular anatomical entity and protein-containing complex. Overall, GO analysis indicated that juvenile hormone analogues mainly affected metabolism-, development-, and regulation-related biological processes in *L. migratoria*.

### 3.4. KEGG Pathway Enrichment Analysis of Differentially Expressed Genes

To identify biological pathways affected by the treatments, KEGG pathway enrichment analysis was performed using DEGs from the ACE-FB vs. HPN-FB and ACE-FB vs. PPF-FB comparison groups.

**Figure 3:**
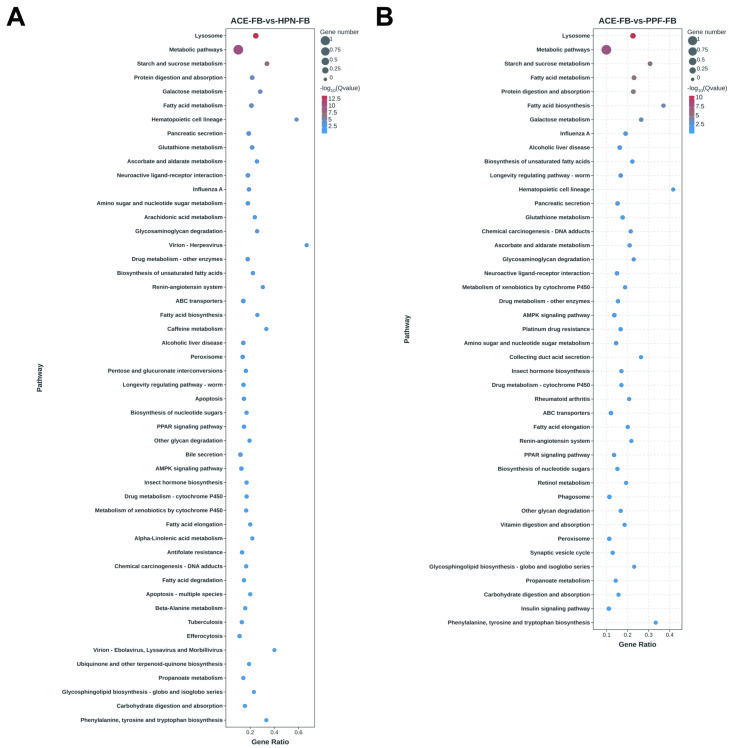
KEGG pathway enrichment analysis of differentially expressed genes (DEGs) in fat body tissues following hydroprene and pyriproxyfen exposure. (**A**) KEGG enrichment analysis of DEGs identified in the ACE-FB vs. HPN-FB comparison. (**B**) KEGG enrichment analysis of DEGs identified in the ACE-FB vs. PPF-FB comparison. Bubble plots show the significantly enriched KEGG pathways identified from DEGs in the two comparison groups. The y-axis represents enriched KEGG pathways, and the x-axis indicates the enrichment factor, calculated as the ratio of DEGs enriched in a given pathway to the total number of annotated genes within that pathway. Bubble size corresponds to the number of DEGs enriched in each pathway, whereas bubble color represents enrichment significance as −log10 (Q value). Red bubbles indicate higher enrichment significance than blue bubbles.

In the ACE-FB vs. HPN-FB comparison (Figure 3A), Metabolic pathways represented the most enriched category and contained the largest number of DEGs. Among the significantly enriched pathways, the Lysosome pathway exhibited the highest enrichment significance, followed by Starch and sucrose metabolism. Additional enriched pathways included Protein digestion and absorption, Galactose metabolism, and Fatty acid metabolism, suggesting that HPN treatment mainly affected lysosome-related functions and carbohydrate metabolism. Similarly, Metabolic pathways remained the dominant enriched category in the ACE-FB vs. PPF-FB comparison (Figure 3B). However, PPF treatment induced broader enrichment in pathways associated with lipid metabolism and detoxification, including Fatty acid biosynthesis, Fatty acid metabolism, Drug metabolism–cytochrome P450, and Metabolism of xenobiotics by cytochrome P450. In addition, energy regulatory pathways such as the AMPK signaling pathway and PPAR signaling pathway were significantly enriched. Compared with the HPN group, the Lysosome pathway displayed relatively lower enrichment significance in the PPF-treated samples.

KEGG enrichment analysis indicated that both treatments primarily disrupted core metabolic pathways, but induced distinct pathway-specific responses. HPN treatment mainly influenced lysosomal function and carbohydrate metabolism, whereas PPF treatment triggered broader alterations in lipid metabolism, detoxification pathways, and energy-related signaling processes.

### 3.5. The Expression of Development-Related Gene Expression Changes in the Fat Body of Locusts

**Figure 4:**
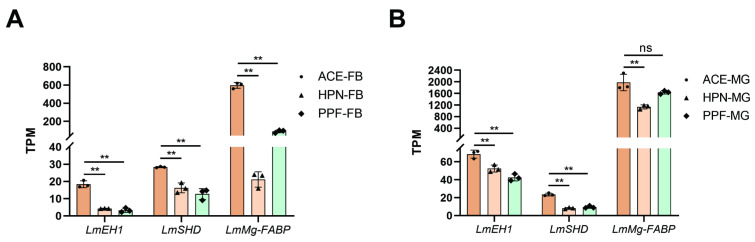
Expression profiles of molting- and development-related genes under insecticide treatments. Transcript abundance (TPM values) of three representative genes (*EH1*, *SHD*, and *Mg-FABP*) was analyzed. (**A**) Comparison of *EH1*, *SHD*, and *Mg-FABP* expression levels in the fat body (FB) among ACE control, HPN-treated, and PPF-treated groups. (**B**) Comparison of *EH1*, *SHD*, and *Mg-FABP* expression levels in the midgut (MG) among ACE control, HPN-treated, and PPF-treated groups. Data are presented as mean ± SD from three independent biological replicates. Statistical significance was determined using Student’s *t*-test (** *p* < 0.01; ns, not significant). *EH1*, juvenile hormone epoxide hydrolase 1-like involved in juvenile hormone metabolism; *SHD*, ecdysone 20-monooxygenase isoform X1 involved in ecdysteroid metabolism; *Mg-FABP*, midgut-enriched fatty acid-binding protein involved in lipid transport and developmental regulation.

To validate the transcriptomic alterations induced by HPN and PPF treatments, the expression profiles of three representative genes involved in molting regulation, hormone signaling, and lipid metabolism were examined (Figure 4A,B). The transcript abundance of *LmMg-FABP* in the midgut was approximately three-fold higher than that in the fat body, indicating a clear midgut-enriched expression pattern.

As shown in Figure 4A (fat body, FB), compared with the ACE control group, all three genes were significantly downregulated following both HPN and PPF treatments (*p* < 0.01, Student’s *t*-test). In the ACE vs. HPN comparison, genes related to juvenile hormone and ecdysteroid regulation, including *EH1* and *SHD*, exhibited markedly reduced transcript levels. In addition, *Mg-FABP*, which is associated with lipid metabolism and developmental regulation, showed the strongest suppression, decreasing from approximately 600 TPM in the control group to ~20 TPM after HPN treatment. A similar expression pattern was observed in the ACE vs. PPF comparison. All three genes were significantly downregulated, with *Mg-FABP* again exhibiting the most pronounced decrease, while *EH1* and *SHD* also showed substantial reductions in transcript abundance.

In contrast, midgut (MG) analysis (Figure 4B) revealed a partially divergent response. HPN treatment significantly reduced the expression of all three genes relative to controls, whereas under PPF exposure, *Mg-FABP* showed no significant change. Accordingly, subsequent functional analyses were focused on the fat body, where transcriptional responses were most robust and consistent.

Collectively, these results demonstrate that both HPN and PPF treatments strongly disrupt the expression of genes involved in molting hormone signaling, ecdysteroid biosynthesis, and lipid metabolism-related developmental processes. These transcriptional changes are consistent with the KEGG enrichment results, which revealed significant perturbations in lipid metabolism and hormone signaling pathways following insecticide treatment.

### 3.6. LmMg-FABP Is Conserved in Orthopteran Insects and Regulates Lipid Accumulation in the Fat Body

**Figure 5:**
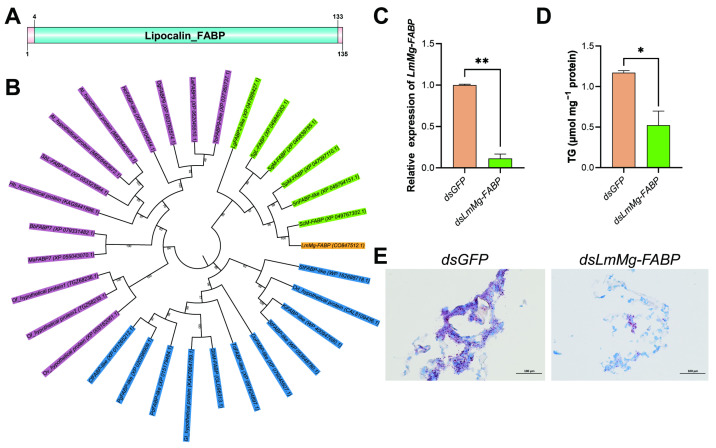
Evolutionary conservation and functional characterization of LmMg-FABP in lipid metabolism. (**A**) Schematic representation of the predicted three-dimensional structure of LmMg-FABP. (**B**) Phylogenetic analysis of FABPs from representative insect species, constructed using the neighbor-joining method, highlighting the evolutionary relationship of LmMg-FABP. Different colors represent different insect species or groups. Bootstrap values (%) are indicated at the nodes. (**C**) RNA interference (RNAi)-mediated silencing efficiency of *LmMg-FABP* in the fat body (FB) following dsRNA injection. (**D**) Triglyceride (TG) levels in the FB after treatment with *dsGFP* or *dsLmMg-FABP*. (**E**) Effects of *LmMg-FABP* knockdown on lipid accumulation in the fat body of *Locusta migratoria* nymphs, as assessed by Oil Red O staining. Red staining indicates lipid droplets, and stronger red staining corresponds to higher lipid accumulation. Data are presented as mean ± SD from three independent biological replicates. Statistical significance was determined using Student’s *t*-test (* *p* < 0.05, ** *p* < 0.01).

The amino acid sequence of LmMg-FABP was identified from the *Locusta migratoria* transcriptome database. To further validate the sequence, we performed RT-PCR amplification followed by Sanger sequencing, which confirmed the accuracy of the full-length coding sequence (Supplementary Figure S2A,B). Conserved domain analysis revealed a typical Lipocalin/FABP domain, confirming that LmMg-FABP belongs to the fatty acid-binding protein family (Figure 5A and Figure S2C). Phylogenetic analysis further showed that LmMg-FABP (CO847512.1) clusters closely with ScM-FABP from *Schistocerca cancellata* (XP049767302.1), indicating strong evolutionary conservation of FABPs among orthopteran insects (Figure 5B).

The biological function of *LmMg-FABP* was investigated using RNA interference (RNAi) in nymphs. RT-qPCR analysis of the fat body at 48 h post-injection confirmed that *dsLmMg-FABP* treatment significantly reduced *LmMg-FABP* transcript levels compared with the control group, demonstrating effective gene silencing and validating RNAi efficiency for subsequent functional analyses (Figure 5C and Figure S2D). In addition, triglyceride (TG) levels in the fat body were measured after RNAi treatment, and as shown in Figure 5D, TG content was significantly reduced compared with the control group.

To further examine the involvement of *LmMg-FABP* in lipid metabolism, Oil Red O staining was conducted on cryosections of nymphal fat bodies following RNAi treatment. Compared with the control group, *dsLmMg-FABP*-treated locusts displayed substantially weaker lipid staining signals, indicating reduced neutral lipid accumulation in the fat body (Figure 5E). These findings suggest that *LmMg-FABP* plays an important role in regulating lipid accumulation and lipid homeostasis in the locust fat body.

### 3.7. Ligand-Binding Properties of LmMg-FABP in L. migratoria

**Figure 6:**
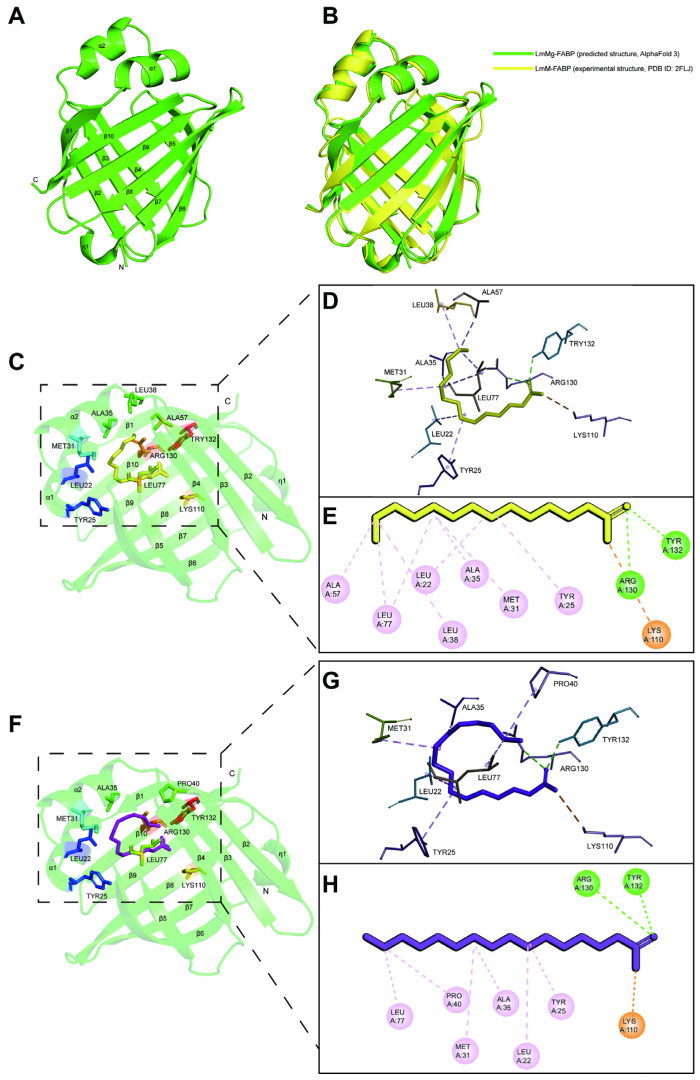
Structural characterization of LmMg-FABP and its fatty acid-binding pocket. (**A**) Predicted three-dimensional structure of LmMg-FABP. (**B**) Structural superposition of LmMg-FABP with LmM-FABP, highlighting conserved folding patterns. (**C**,**F**) Overall view of the fatty acid-binding pocket. The ligands myristic acid (MYR) and palmitic acid (PLM) are shown in stick representation, with MYR colored yellow and PLM purple. The protein backbone is displayed as a green cartoon model. (**D**,**G**) Close-up three-dimensional views of the fatty acid-binding pocket, illustrating the detailed ligand–protein interactions. (**E**,**H**) Two-dimensional schematic representations of the binding pocket, showing interactions between ligands and surrounding amino acid residues. MYR and PLM are positioned within the binding cavity, and key residues are labeled in black. Pink dashed lines indicate hydrophobic interactions, green dashed lines represent hydrogen bonds, and orange dashed lines indicate electrostatic interactions between the ligands and the protein.

The three-dimensional structure of LmMg-FABP was predicted using AlphaFold 3, revealing a hydrophobic ligand-binding cavity (Figure 6A). The predicted template modeling (pTM) score was 0.91, suggesting a high-confidence and reliable global structural model (Supplementary Figure S3). Similar to FABPs from humans, migratory locusts, desert locusts, fruit flies, tobacco hornworms, and zebrafish, LmMg-FABP exhibits a typical α/β fold arrangement (Figure 6B and Figure S4A,B), which is a conserved structural feature of the FABP family. The secondary structure elements are arranged in the order η1-α1–α2–β1–β2–β3–β4–β5–β6–β7–β8–β9–β10. The protein core consists of a ten-stranded β-sheet (strand order 3–2–1–10–9–8–7–6–5–4) covered by two α-helices and a short η-helix, forming a large hydrophobic cavity capable of accommodating fatty acid molecules.

Comparative structural analysis showed that LmMg-FABP shares moderate sequence identity with FABPs from diverse vertebrate and insect species, with amino acid identities ranging from 21% to 43% (Table 2, Supplementary Figure S4A). Despite the relatively low sequence conservation, the predicted structure of LmMg-FABP exhibited high structural similarity to previously characterized FABPs, as indicated by the low RMSD values (1.1–2.3 Å). These results suggest that the overall FABP fold is highly conserved during evolution, whereas the primary amino acid sequences have undergone substantial divergence among species.

Among the compared proteins, the highest structural similarity was observed with the *Locusta migratoria* M-FABP structure (PDB: 2FLJ; RMSD = 1.1 Å), while lower similarity was detected with the zebrafish homolog (PDB: 3ELZ; RMSD = 2.3 Å). Insect FABPs from *Schistocerca gregaria*, *Drosophila melanogaster*, and *Manduca sexta* also displayed conserved structural architectures despite relatively modest sequence identities (35–43%). These findings indicate that the conserved tertiary structure of FABPs may be more critical for maintaining lipid-binding function than strict conservation of amino acid sequence.

Furthermore, variations in amino acid composition among species were mainly distributed in loop regions and peripheral residues, whereas residues associated with the hydrophobic fatty acid-binding cavity were comparatively conserved. Such structural conservation may preserve the ability of FABPs to bind fatty acid ligands, while sequence divergence could contribute to species-specific substrate selectivity and functional adaptation.

The Ramachandran plot indicated that 99.2% of residues were located in favored regions, and 100% were within allowed regions, with no outliers (Supplementary Figure S5). In addition, 97.04% of residues exhibited 3D–1D scores greater than 0.1 in Verify3D analysis (Supplementary Figure S6), supporting the reliability of the predicted model. Pocket analysis identified multiple hydrophobic residues lining the binding cavity, including MET18, PHE21, LEU22, TYR25, VAL27, MET31, ALA35, LEU38, PRO40, THR42, GLN53, THR55, ALA57, ARG60, VAL62, THR64, GLU74, THR75, THR76, LEU77, ASP78, GLN96, GLN98, PHE100 and VAL106, which may participate in fatty acid binding (Supplementary Figure S7).

Molecular docking analysis revealed that both MYR and PLM were stably accommodated within the hydrophobic cavity of LmMg-FABP and displayed highly similar binding conformations (Figure 6C–H). In both complexes, the aliphatic chains of the ligands were surrounded by multiple hydrophobic residues, including LEU22, TYR25, ALA35, MET31, and LE77, indicating that hydrophobic interactions constitute the primary driving force for ligand recognition and stabilization. In addition, the terminal polar groups of both ligands formed hydrogen bonds with conserved polar residues, particularly TYR132 and ARG130, while also engaging in electrostatic interactions with LYS110, further enhancing the stability of the protein–ligand complexes. Despite their similar binding modes, subtle differences in residue interactions were observed between the two ligands. MYR additionally interacted with ALA57 and LEU38, which were not observed in the PLM complex. These differences suggest that LmMg-FABP exhibits a certain degree of ligand selectivity and conformational adaptability toward structurally distinct fatty acids.

Overall, these findings indicate that LmMg-FABP recognizes fatty acid ligands through a cooperative mechanism involving a conserved hydrophobic binding environment together with specific polar interactions at the ligand head group. The flexible accommodation of different fatty acids within the binding cavity suggests that LmMg-FABP possesses the ability to recognize and bind fatty acid ligands with different chain lengths, highlighting its structural adaptability toward diverse lipid substrates.

## 4. Discussion

The present study provides new insights into the mechanisms by which juvenile hormone analogues (JHAs), including pyriproxyfen and hydroprene, disrupt insect physiology by linking endocrine interference with metabolic reprogramming. Consistent with their function as mimics of endogenous juvenile hormone (JH), both compounds significantly affected hormonal homeostasis and were associated with pronounced developmental and physiological alterations.

Our gene expression analyses revealed that key genes involved in 20-hydroxyecdysone (20E) biosynthesis and signaling, including *EH1* and *SHD*, were significantly altered following JHA exposure, suggesting that ecdysteroid-related developmental pathways are responsive to JHA treatment. These results are consistent with a model in which JHAs may interfere with the balance between JH and 20E signaling, a conserved endocrine interaction essential for insect molting and metamorphosis. Similar antagonistic interactions between these hormonal pathways have been reported in multiple insect species, where JHA exposure leads to abnormal development and developmental delay.

Beyond canonical endocrine disruption, our data further indicate that JHAs induce a systemic imbalance between JH and ecdysteroid regulatory networks. The observed alterations in hormone-associated gene expression suggest an antagonistic interaction between these endocrine axes, whereby persistent JH signaling is associated with suppression of 20E biosynthesis and downstream signaling. This is consistent with earlier findings in *Bombyx mori* and other insect species, where pyriproxyfen exposure inhibits ecdysteroid signaling and results in abnormal metamorphosis and developmental delay [13,14]. Similar endocrine-disrupting effects have also been reported for hydroprene, which prolongs larval development and impairs metamorphic progression [5,6]. Collectively, these findings reinforce the conserved role of JHAs in disrupting hormonal homeostasis across insect taxa.

Importantly, transcriptomic evidence from this study further reveals that JHAs exert profound effects on lipid metabolic networks, indicating that metabolic dysregulation is an integral component of their toxicity rather than a secondary consequence. This is supported by consistent downregulation of lipid metabolism-related genes and is in agreement with recent metabolomic and lipidomic studies demonstrating that pyriproxyfen and related insect growth regulators significantly alter fatty acid composition and lipid metabolic pathways [17], as well as induce tissue-specific lipid remodeling in aquatic invertebrates such as *Daphnia magna* [18]. These findings support an emerging model in which JHA toxicity extends beyond endocrine disruption to involve broad metabolic reprogramming.

A central finding of this study is the involvement of *Mg-FABP*, in the response to JHA exposure. *FABPs* function as intracellular lipid chaperones responsible for fatty acid transport and homeostasis, and are essential for energy metabolism, development, and stress adaptation in insects. In this study, *Mg-FABP* showed the most pronounced and consistent downregulation under both treatments, suggesting that it represents a key regulatory node in JHA-induced metabolic disruption. Given the critical role of *FABP*-mediated lipid trafficking in physiological processes such as flight and diapause-associated lipid storage, its dysregulation likely contributes significantly to the observed metabolic imbalance. Although the present study focused on a biologically effective dose identified through preliminary range-finding experiments, future dose–response studies will be valuable for further characterizing the relationship between JHA concentration and *LmMg-FABP* expression and for defining the sensitivity of this response.

To validate transcriptomic findings and further explore lipid regulatory mechanisms, we analyzed the structural and ligand-binding properties of LmMg-FABP. Structural comparison revealed that LmMg-FABP shares a conserved tertiary structure with FABP homologs despite relatively low sequence identity, indicating strong evolutionary constraints on the lipid-binding fold. Notably, residues forming the hydrophobic binding cavity were more conserved than peripheral regions, suggesting preserved functional constraints in ligand recognition. To further evaluate the reliability of the structural modeling approach, we first used AlphaFold 3 to predict the structures of the LmM-FABP–oleic acid (OLA) and MsFABP2–palmitate (PLM) complexes and compared them with previously reported experimentally determined structures (Supplementary Figures S8A,B and S9A,B). The predicted models showed excellent agreement with the experimental structures, with RMSD values of 0.330 Å and 0.315 Å, respectively. Based on this validation, we further applied AlphaFold 3 to predict the structures of LmMg-FABP in complex with MYR and PLM. The resulting models showed consistently high confidence scores, indicating reliable structural predictions (Supplementary Figures S10 and S11).

Molecular docking analyses demonstrated that fatty acids (MYR and PLM) can be stably accommodated within the hydrophobic pocket of LmMg-FABP. Binding was mainly driven by hydrophobic interactions within the ligand-binding cavity of LmMg-FABP, where residues such as LEU22, TYR25, ALA35, MET31, and LEU77 collectively stabilized ligand accommodation. In addition, hydrogen bonding and electrostatic interactions involving conserved residues, including TYR132, ARG130, and LYS110, further contributed to binding specificity and complex stability. Subtle ligand-dependent differences were observed, suggesting that LmMg-FABP possesses conformational flexibility enabling recognition of structurally distinct fatty acids. However, it should be noted that AlphaFold 3-predicted complex structure provide predictive evidence of binding potential rather than direct biochemical confirmation. Future studies combining additional mutagenesis and biochemical binding assays will be necessary to further validate the roles of these residues and to clarify the molecular mechanisms underlying Mg-FABP-mediated fatty acids transport in Orthopteran insects.

## 5. Conclusions

In summary, this study provides evidence fora multi-layered mechanism underlying the toxicity of juvenile hormone analogues (JHAs), including pyriproxyfen and hydroprene, in insects. Our findings suggest that JHA exposure may disrupt the endocrine equilibrium between juvenile hormone and 20-hydroxyecdysone signaling, as evidenced by altered expression of key genes involved in ecdysteroid biosynthesis and signaling. This hormonal imbalance is associated with impaired developmental progression and physiological dysfunction.

Beyond endocrine disruption, we further show that JHAs induce extensive reprogramming of lipid metabolic networks, indicating that metabolic dysregulation likely contributes to their mode of action. In particular, the pronounced downregulation of Mg-FABP suggests that fatty acid-binding proteins may act as potential mediators linking hormonal signaling to lipid homeostasis. Structural and docking analyses of LmMg-FABP further support a conserved lipid-binding architecture and ligand flexibility, which may be consistent with its role in fatty acid transport and metabolic regulation.

Collectively, our results suggest a unified model in which JHAs are associated with toxicity effects through coordinated disruption of endocrine signaling and lipid metabolic pathways, ultimately impairing insect development and energy homeostasis. These findings not only contribute to a better understanding of the JHA mode of action at the molecular level but also highlight FABP-associated lipid transport as a potential target for ecological risk assessment and the design of more selective insect growth regulators.

## Figures and Tables

**Table 1:** Summary of RNA-seq read mapping statistics based on the *Locusta migratoria* reference genome.

Sample	Total	Unmapped (%)	Unique Mapped (%)	Multiple Mapped (%)	Total Mapped (%)
ACE-FB	44,286,284 ± 8,039,391.81	13.54 ± 1.22	74.26 ± 1.51	12.1 ± 0.35	86.46 ± 1.22
ACE-MG	40,933,388 ± 8,188,046.68	17.10 ± 1.02	71.18 ± 0.20	11.71 ± 0.85	82.90 ± 1.00
HPN-FB	41,303,506.67 ± 3,418,178.03	12.62 ± 0.60	77.23 ± 0.20	10.14 ± 0.60	87.38 ± 0.60
HPN-MG	40,078,066 ± 2,394,117.20	17.97 ± 1.23	70.8 ± 1.066	11.16 ± 0.18	82.03 ± 1.23
PPF-FB-	38,109,086 ± 5,677,214.05	12.24 ± 0.81	77.78 ± 0.42	9.98 ± 0.39	87.66 ± 0.66
PPF-MG	40,245,138.67 ± 4,222,940.23	18.01 ± 0.67	71.26 ± 0.86	10.73 ± 0.20	81.99 ± 0.67

“Total” indicates the number of clean reads obtained after quality filtering; “Unmapped (%)” represents the number and proportion of reads that could not be aligned to the reference genome; “Unique Mapped (%)” indicates the number and proportion of reads uniquely mapped to the reference genome; “Multiple Mapped (%)” represents the number and proportion of reads mapped to multiple genomic locations; “Total Mapped (%)” indicates the total number and proportion of reads successfully aligned to the reference genome.

**Table 2:** Proteins with similar structures to LmMg-FABP.

Organism	PDB	RMSD ^a^	Identities	Method	Resolution	Description
*Locusta migratoria*	2FLJ	1.1 Å	38%	NMR	-	M-FABP
*Schistocerca gregaria*	1FTP	1.3 Å	39%	X-ray	2.20 Å	M-FABP
*Homo sapiens*	7FWV	1.4 Å	34%	X-ray	1.45 Å	A-FABP
*Drosophila melanogaster*	5GGE	1.5 Å	43%	X-ray	1.86 Å	M-FABP
*Manduca sexta*	1MDC	1.6 Å	35%	X-ray	1.75 Å	FABP2
*Danio rerio*	3ELZ	2.3 Å	21%	X-ray	2.20 Å	FABP6

^a^ RMSD (root-mean-square deviation) represents the average positional deviation of corresponding backbone Cα atoms between two structurally aligned proteins and is commonly used to evaluate structural similarity.

## Data Availability

All data from the study are available in the Supporting Material or upon demand.

## Supplementary Materials

The following supporting information can be downloaded at: https://www.mdpi.com/article/10.3390/insects17070664/s1, Figure S1: Effects of juvenile hormone analogs on *Locust mortality* and experimental workflow for transcriptome analysis; Figure S2: Cloning, sequencing validation, conserved domain analysis and functional characterization of *LmMg-FABP*; Figure S3: 3D structural prediction of LmMg-FABP; Figure S4: Alignment of FABP domain sequences from several species; Figure S5: Validation of LmMg-FABP 3D structure via MolProbity Ramachandran analysis; Figure S6: Validation of LmMg-FABP 3D structure via Verify3D analysis; Figure S7: Schematic showing the hydrophobic residues on the pocket of LmMg-FABP domain; residues surrounding the cavity are highlighted with green color; Figure S8: Alignment analysis of the LmM-FABP–oleic acid (OLA) complex; Figure S9: Alignment analysis of the MsFABP2–PLM complex; Figure S10: The LmMg-FABP-MYR complex was predicted using AlphaFold 3; Figure S11: The LmMg-FABP–PLM complex was predicted using AlphaFold 3; Table S1: Double-stranded RNA primers; Table S2: Sequences of the primers used for real time PCR analyzes; Table S3: Summary of raw and clean RNA-seq reads after quality filtering; Data S1–S3 can be downloaded at https://doi.org/10.6084/m9.figshare.32788740, Data S1: RNA-sequencing summary and transcriptomic analysis results, including Reads Stat, Alignment Stat, New Gene Annotation, Gene Assessment, Expression Stat, Sample Relation, and Group Diff Expression; Data S2: Reference genome sequence and corresponding annotation files, including genomic DNA sequence, GFF3 annotation file, CDS sequences, and predicted protein sequences of *Locusta migratoria*; Data S3: Validated full-length *LmMg-FABP* sequence and associated sequence verification dataset.
