# Supplementary material for: Juvenile Hormone Analogues Reduce the Expression of a Fatty Acid-Binding Protein Involved in Lipid Accumulation in the Migratory Locust Locusta migratoria
Source: Insects. 2026 Jun 25;17(7):664. doi: 10.3390/insects17070664 (PMC13411818; doi:10.3390/insects17070664)
Supplement: Supplementary file 1 [file insects-17-00664-s001.zip › insects-4347597-supplementary.pdf]

Supplementary information：

Juvenile Hormone Analogues Reduce the Expression of a Fatty Acid-Binding Protein Involved in Lipid Accumulation in the Migratory Locust *Locusta migratoria*

Tian Miao[1], Zige Wang[1], Min Peng[1], Jinchao Chen[1], Dengbo Li[1] and Yuemin Ma [1,2*]

[1]College of Life Science, Shanxi University, Taiyuan 030006, Shanxi, P. R. China;

[2]School of Synthetic Biology, Shanxi University, Taiyuan 030006, Shanxi, P. R. China; ymma@sxu.edu.cn

[*]Correspondence: Correspondence and requests for materials should be addressed to yuemin ma (E-mail address: ymma@sxu.edu.cn); Tel.:13007102085

Tian Miao: miaotian@sxu.edu.cn

Zige Wang: wangzige1@sxu.edu.cn

Min Peng: pengmin@sxu.edu.cn

Jinchao Chen: chenjinchao@sxu.edu.cn

Dengbo Li: lidengbo@sxu.edu.cn

Yuemin ma (corresponding author): ymma@sxu.edu.cn

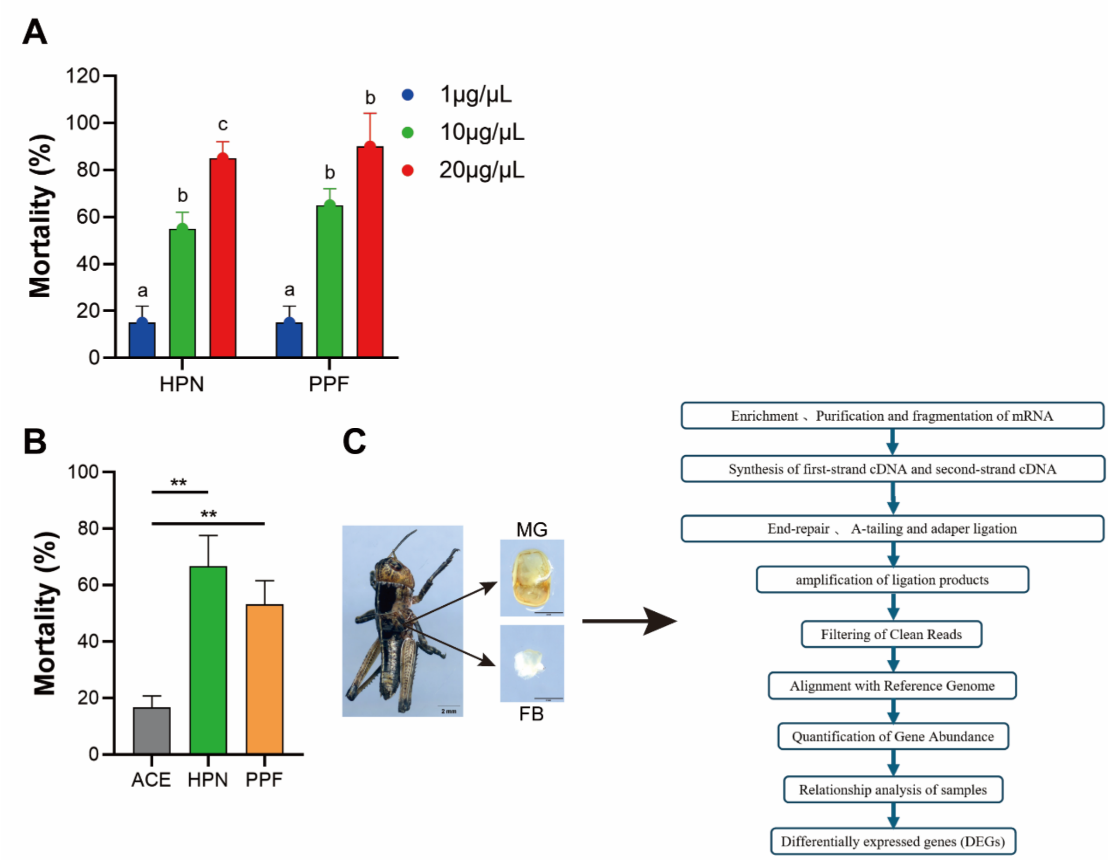

Figure. S1. Effects of juvenile hormone analogs on locust mortality and experimental workflow for transcriptome analysis. (A) Preliminary dose-response assay of locust nymphs exposed to different concentrations (1, 10, and 20 μg/μL) of hydroprene (HPN) and pyriproxyfen (PPF). Mortality was recorded after treatment to determine the optimal concentration for subsequent experiments. (B) Mortality (%) of locusts following exposure to acetone (ACE, solvent control), HPN, and PPF at 10 μg/μL. Data are presented as mean ± SD from independent biological replicates. (C) Schematic workflow of transcriptome sequencing and analysis using fat body (FB) and midgut (MG) tissues dissected from locusts.

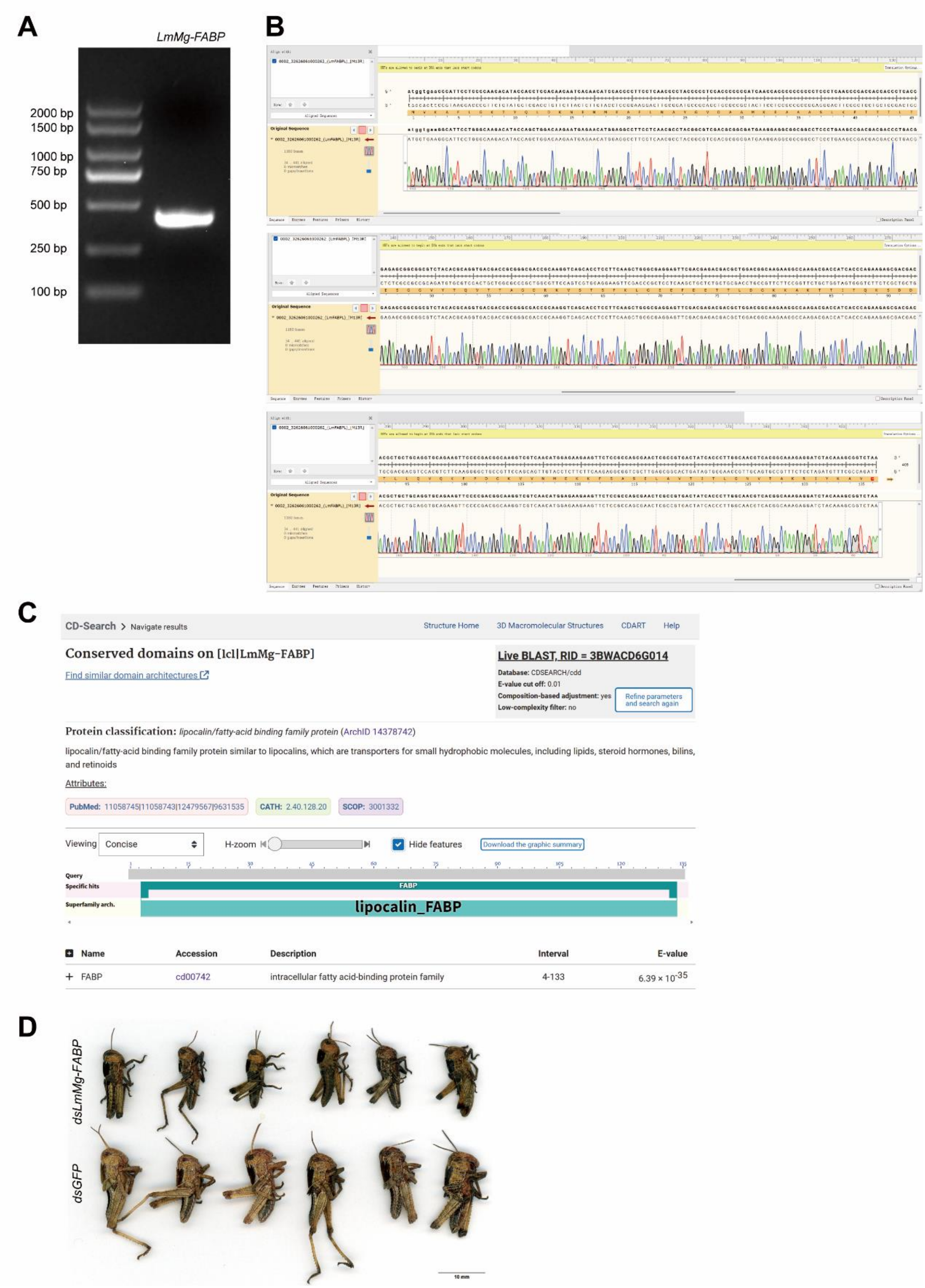

Figure. S2. Cloning, sequencing validation, conserved domain analysis and functional characterization of *LmMg-FABP*. (A) PCR amplification of the *LmMg-FABP* coding sequence. (B) The sequencing results confirmed the accuracy of

the cloned sequence. (C) Conserved domain analysis of *LmMg-FABP*. (D) Phenotypic effects on growth and development in *L. migratoria* nymphs after dsRNA injection.

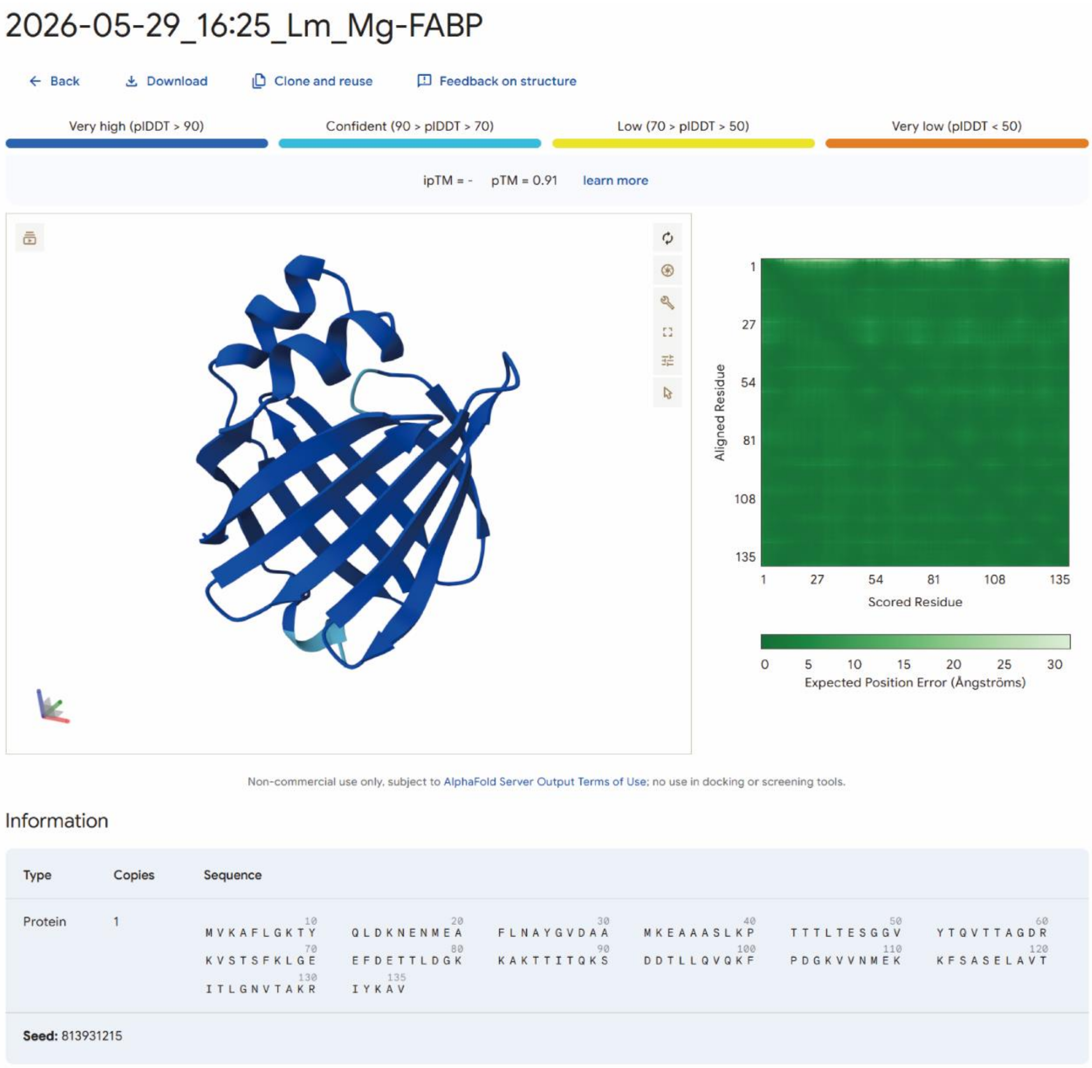

Figure. S3. 3D Structural Prediction of LmMg-FABP.

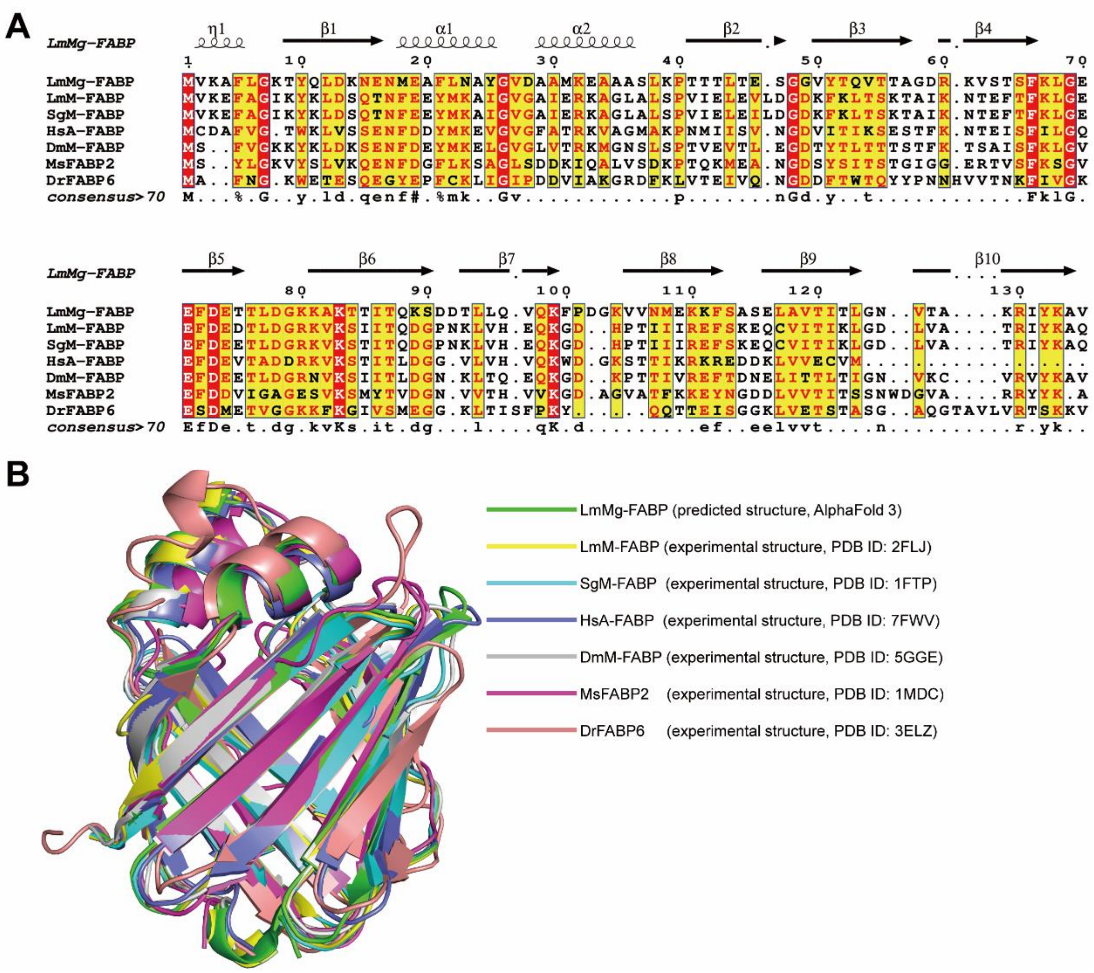

Figure. S4. Alignment of FABP domain sequences from several species.

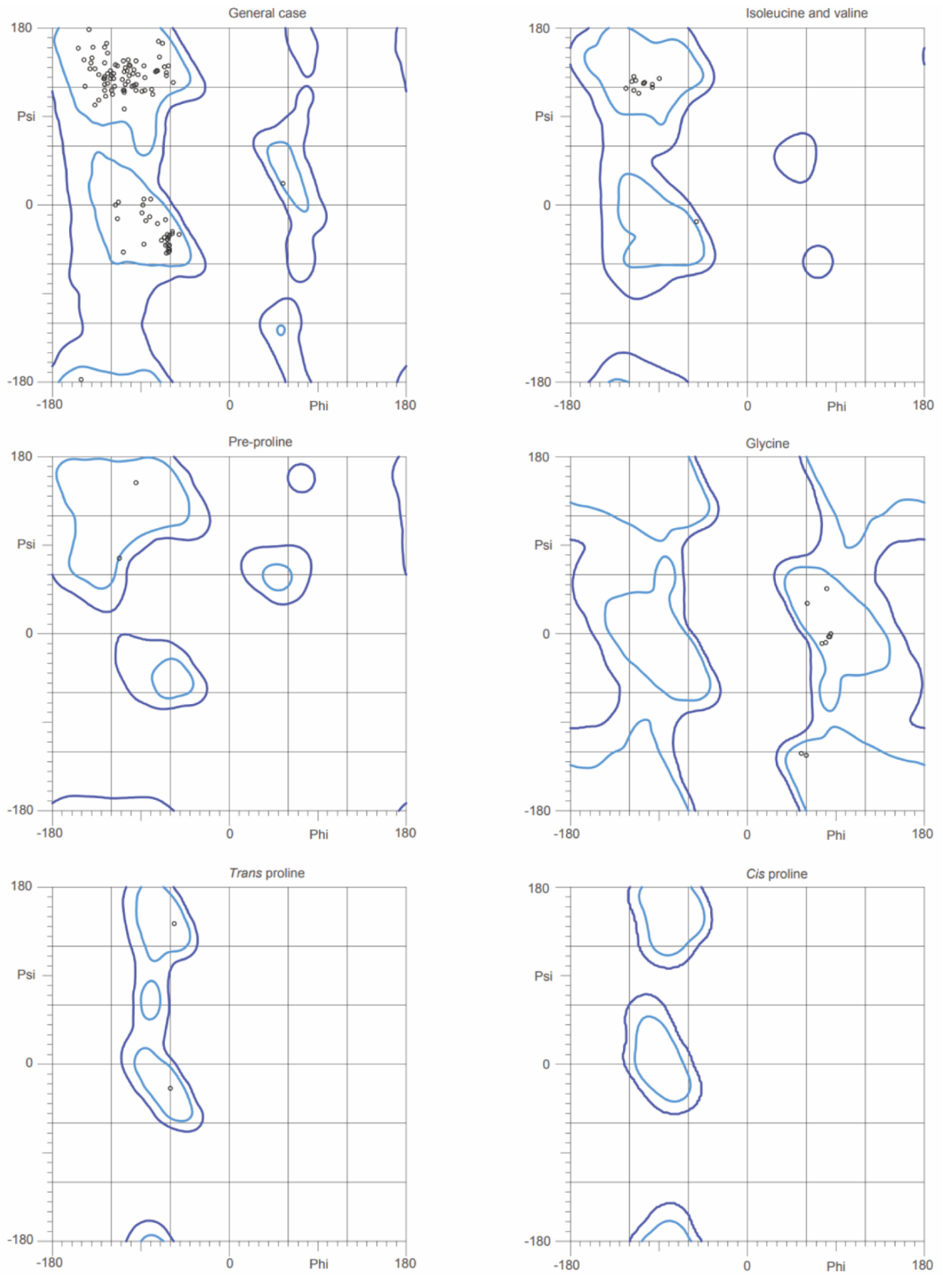

Figure. S5. Validation of LmMg-FABP 3D Structure via MolProbity Ramachandran Analysis. The constructed 3D model of LmMg-FABP was evaluated using MolProbity Ramachandran analysis to assess the stereochemical quality and reliability of the predicted structure, ensuring that most residues fall within favored and allowed regions.

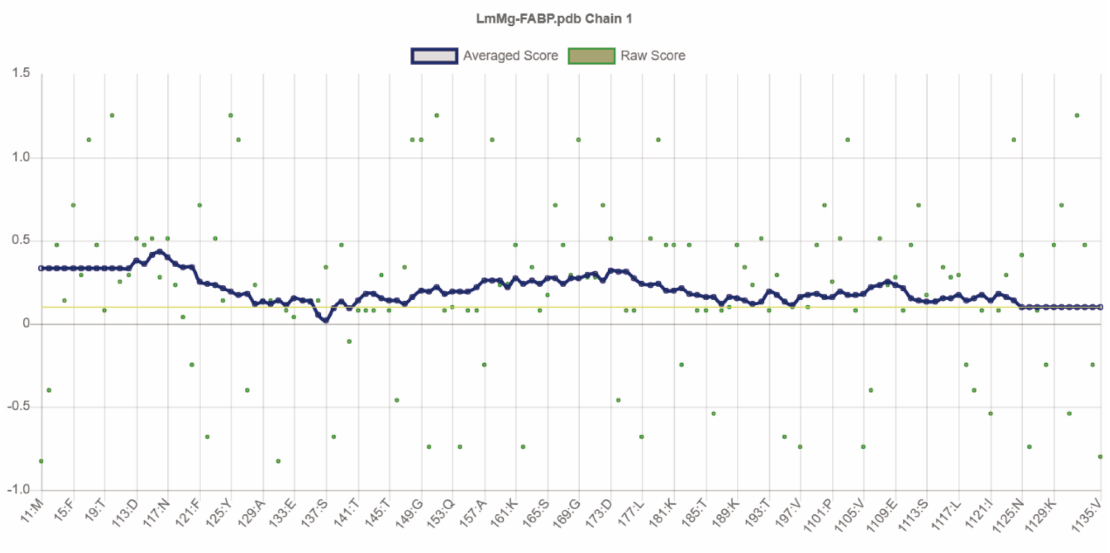

Figure. S6. Validation of LmMg-FABP 3D Structure via Verify 3D analysis. This complementary assessment confirmed that the overall folding and residue environments were consistent with expected patterns, providing additional confidence in the model's suitability for downstream molecular docking and functional studies.

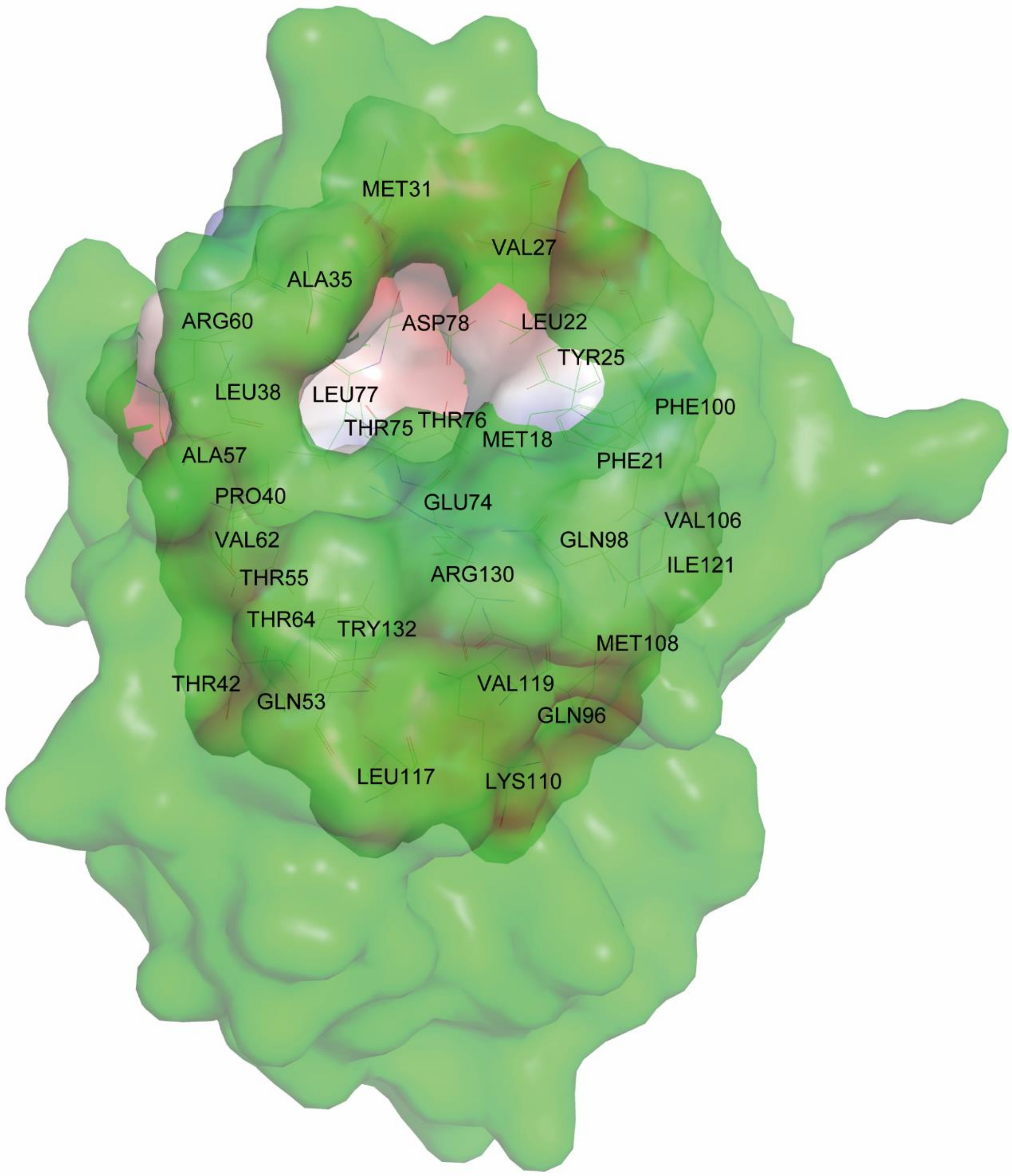

Figure. S7. Schematic showing the hydrophobic residues on the pocket of LmMg-FABP domain, residues surrounding the cavity are highlighted with green color.

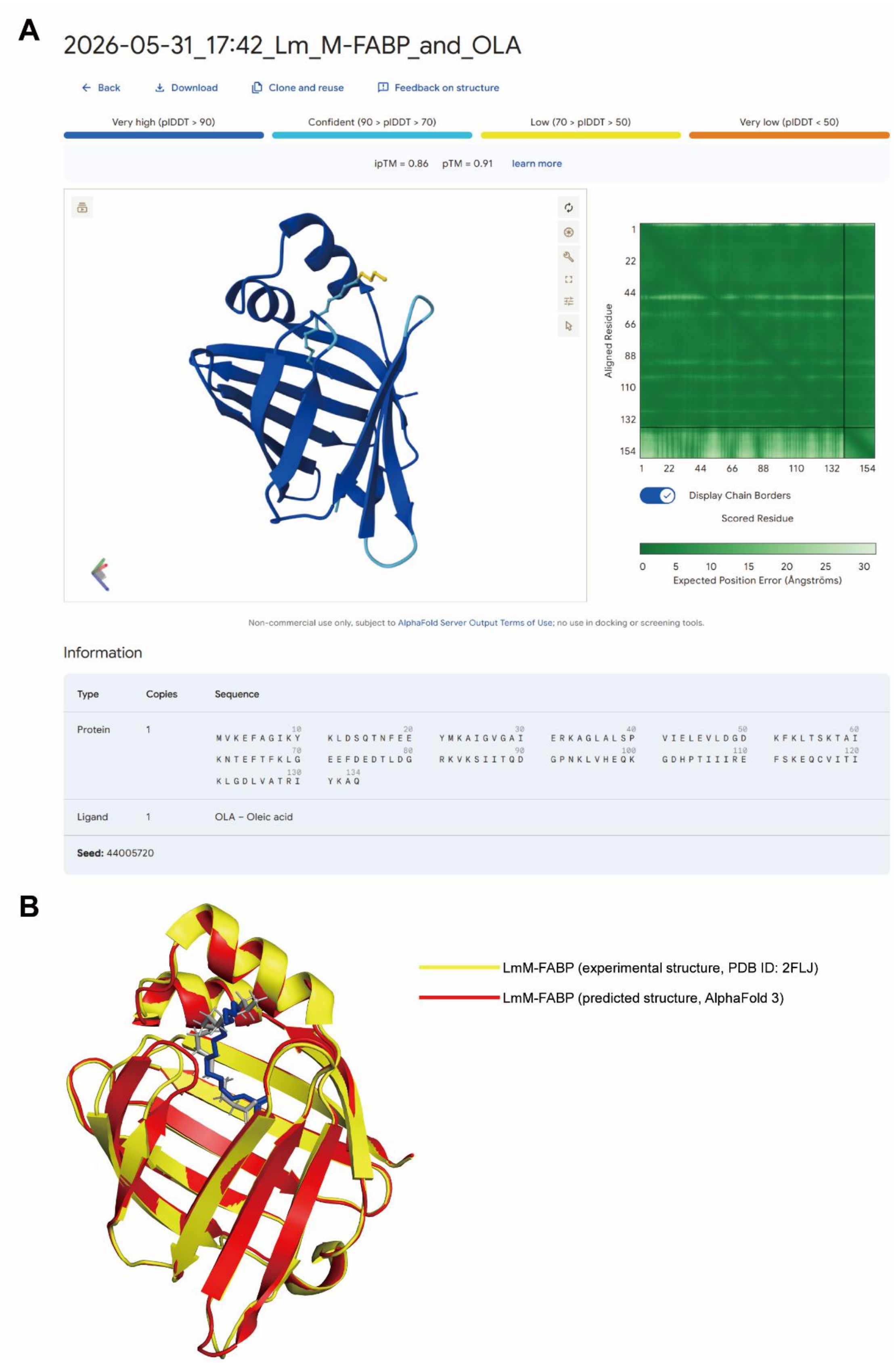

Figure. S8. Alignment analysis of the LmM-FABP–oleic acid (OLA) complex. (A) Predicted 3D structure of the LmM-FABP–OLA complex generated using

AlphaFold 3. (B) Structural alignment of the AlphaFold 3-predicted LmM-FABP–OLA complex with the experimentally determined LmM-FABP–OLA complex (PDB ID: 2FLJ).

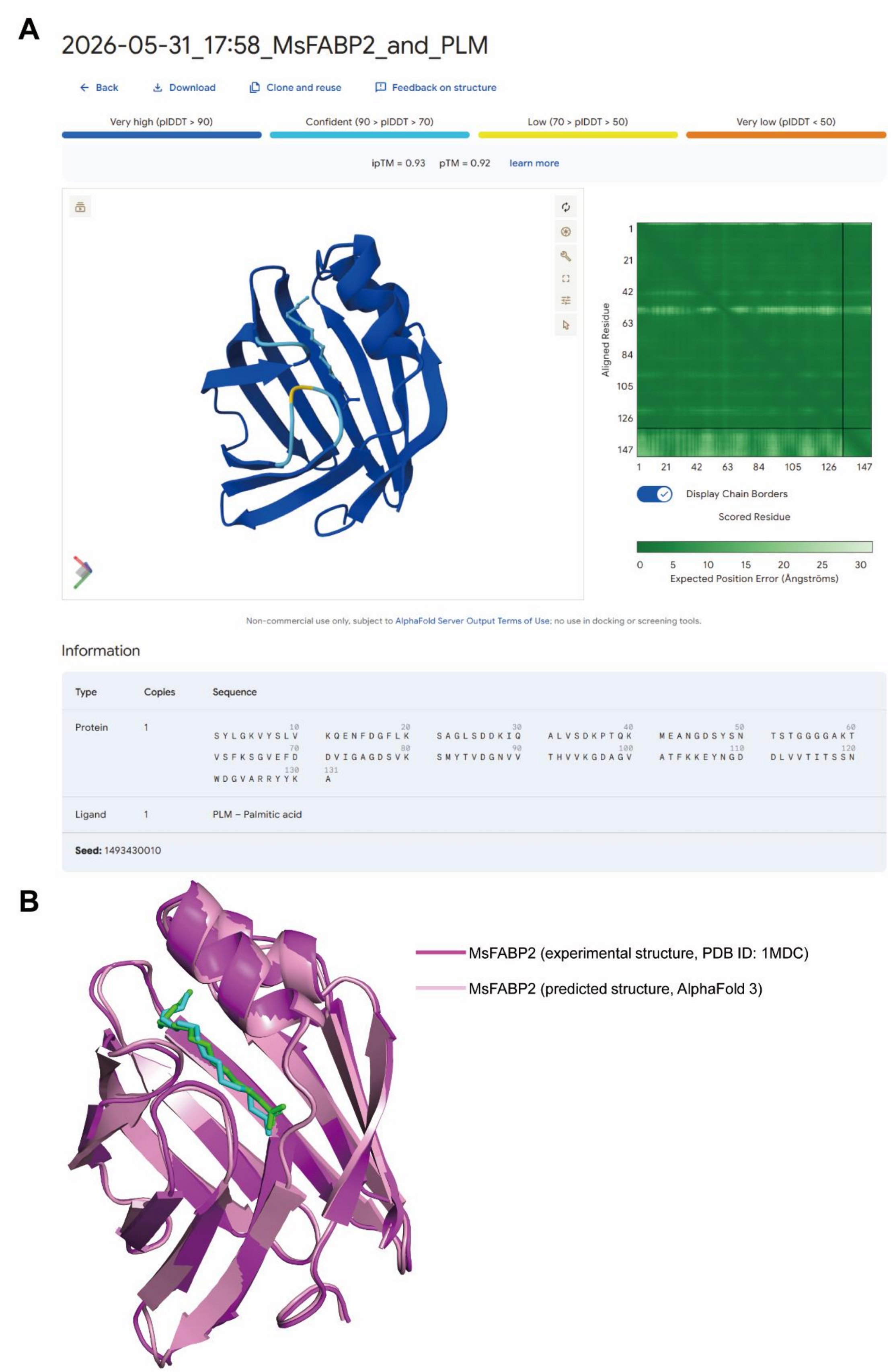

Figure. S9. Alignment analysis of the MsFABP2–PLM complex. (A) Predicted

3D structure of the MsFABP2–PLM complex generated using AlphaFold 3. (B) Structural alignment of the AlphaFold 3-predicted MsFABP2–PLM complex with the experimentally determined MsFABP2–PLM complex (PDB ID: 1MDC).

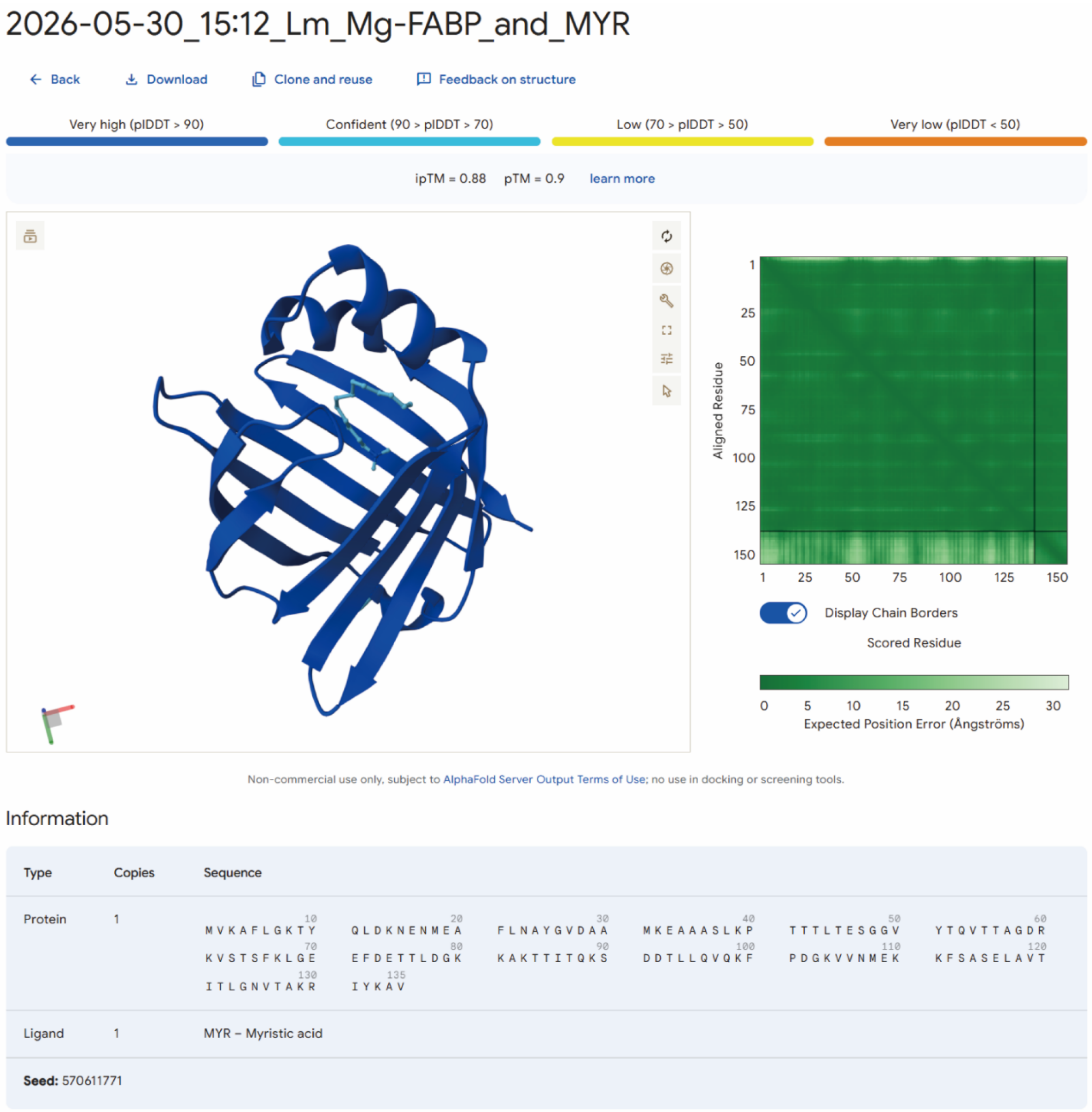

Figure. S10. The LmMg-FABP–MYR complex was predicted using AlphaFold 3.

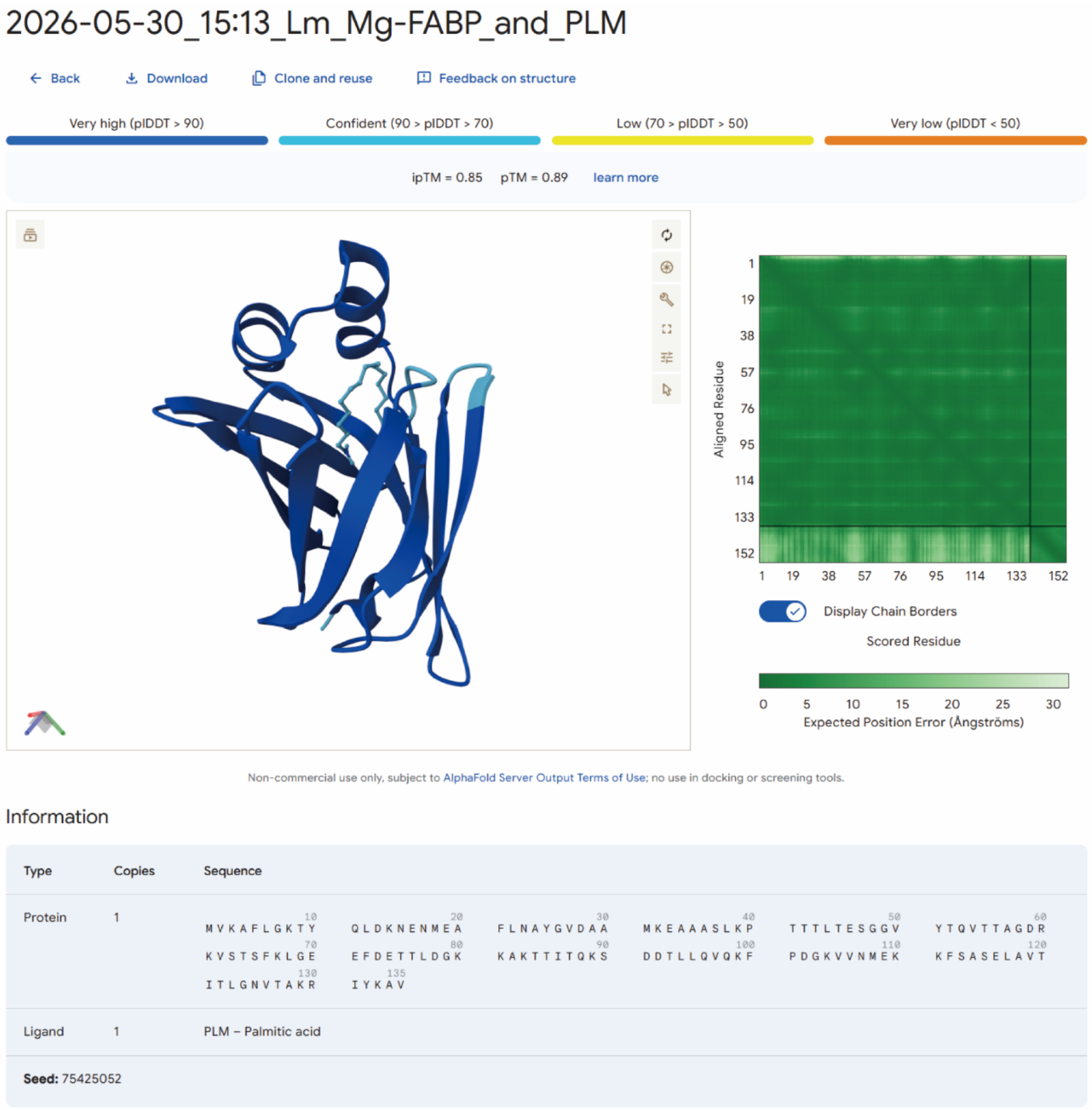

Figure. S11. The LmMg-FABP–PLM complex was predicted using AlphaFold 3.

Table. S1 Double-stranded RNA primers

Primer	Primer sequences (5′-3′)
dsGFP-F	TAATACGACTCACTATAGGGTGGAGAGGGTGAAGG
dsGFP-R	TAATACGACTCACTATAGGGGGGCAGATTGTGTGGAC
dsLmMg-FABP-F	TAATACGACTCACTATAGGGCTTCCTCAACGCCTACGG
dsLmMg-FABP-R	TAATACGACTCACTATAGGGGTCCAGCGTCGTCTCGTC

Table. S2 Sequences of the primers used for real time PCR analyzes

Primer	Primer sequences (5′-3′)
EF-1α-F	AGCCCAGGAGATGGGTAAAG
EF-1α-R	CTCTGTGGCCTGGAGCATC
LmMg-FABP-F	CCAAGACGACCATCACCC
LmMg-FABP-R	CGAGTTCGCTGGCGGAGA

Table S3. Summary of raw and clean RNA-seq reads after quality filtering

Sample	RawDatas	CleanData (%)	Adapter (%)	LowQuality (%)	polyA (%)	N (%)
ACE-FB-1	46050166	45526290 (98.86%)	343224 (0.75%)	179956 (0.39%)	0 (0.00%)	696 (0.00%)
ACE-FB-2	57608288	57119130 (99.15%)	389146 (0.68%)	99858 (0.17%)	0 (0.00%)	154 (0.00%)
ACE-FB-3	40913300	40408712 (98.77%)	336978 (0.82%)	167528 (0.41%)	0 (0.00%)	82 (0.00%)
ACE-MG-1	36497734	36101524 (98.91%)	234820 (0.64%)	161336 (0.44%)	0 (0.00%)	54 (0.00%)
ACE-MG-2	41943488	41486962 (98.91%)	268402 (0.64%)	188042 (0.45%)	0 (0.00%)	82 (0.00%)
ACE-MG-3	53241068	52543282 (98.69%)	579550 (1.09%)	116764 (0.22%)	0 (0.00%)	1472 (0.00%)
HPN-FB-1	47223610	46676070 (98.84%)	370664 (0.78%)	176128 (0.37%)	0 (0.00%)	748 (0.00%)
HPN-FB-2	44690898	44433612 (99.42%)	142980 (0.32%)	114266 (0.26%)	0 (0.00%)	40 (0.00%)
HPN-FB-3	39895078	39546082 (99.13%)	211484 (0.53%)	137512 (0.34%)	0 (0.00%)	0 (0.00%)
HPN-MG-1	40337792	39987982 (99.13%)	196206 (0.49%)	153534 (0.38%)	0 (0.00%)	70 (0.00%)
HPN-MG-2	40978872	40623222 (99.13%)	243156 (0.59%)	112452 (0.27%)	0 (0.00%)	42 (0.00%)
HPN-MG-3	44556544	44100954 (98.98%)	297348 (0.67%)	158242 (0.36%)	0 (0.00%)	0 (0.00%)
PPF-FB-1	48812830	48344622 (99.04%)	347162 (0.71%)	117966 (0.24%)	0 (0.00%)	3080 (0.01%)
PPF-FB-2	37484166	37139682 (99.08%)	217974 (0.58%)	126468 (0.34%)	0 (0.00%)	42 (0.00%)
PPF-FB-3	38956356	38645062 (99.20%)	174786 (0.45%)	136508 (0.35%)	0 (0.00%)	0 (0.00%)
PPF-MG-1	37826052	37509482 (99.16%)	186696 (0.49%)	129874 (0.34%)	0 (0.00%)	0 (0.00%)
PPF-MG-2	45655422	45198722 (99.00%)	291206 (0.64%)	165494 (0.36%)	0 (0.00%)	0 (0.00%)
PPF-MG-3	45069968	44657596 (99.09%)	226686 (0.50%)	185298 (0.41%)	0 (0.00%)	388 (0.00%)

Raw reads represent the total number of sequencing reads generated from each sample. Clean reads were obtained after removing adapter sequences, low-quality reads, reads containing ambiguous bases (N), and poly(A)-containing reads. The percentages indicate the proportion of reads removed or retained during the filtering process.

Supplementary Data 1: RNA-sequencing summary and transcriptomic analysis results, including Reads Stat, Alignment Stat, New Gene Annotation, Gene Assessment, Expression Stat, Sample Relation, and Group Diff Expression. The sample naming system used in the RNA-seq analysis files is explained as follows: CK corresponds to ACE, HY corresponds to HPN, and PY corresponds to PPF.

Supplementary Data 2: Reference genome sequence and corresponding annotation files, including genomic DNA sequence, GFF3 annotation file, CDS sequences, and predicted protein sequences of *Locusta migratoria*.

Supplementary Data 3: Validated full-length *LmMg-FABP* sequence and associated sequence verification dataset.
